# Fabrication of Dentin-Pulp-Like Organoids Using Dental-Pulp Stem Cells

**DOI:** 10.3390/cells9030642

**Published:** 2020-03-06

**Authors:** Sang Yun Jeong, Soonchul Lee, Woo Hee Choi, Joo Hyun Jee, Hyung-Ryong Kim, Jongman Yoo

**Affiliations:** 1 Department of Microbiology and CHA Organoid Research Center, CHA University School of Medicine, Gyeonggi-do 13488, Korea; 7023jsy@chauniv.ac.kr (S.Y.J.); whchoi1204@gmail.com (W.H.C.); Joohyun@chauniv.ac.kr (J.H.J.); 2 Department of Orthopaedic Surgery, CHA Bundang Medical Center, CHA University School of Medicine, Gyeonggi-do 13496, Korea; lsceline78@gmail.com; 3 College of Dentistry, Dankook University, Cheonan 31116, Korea

**Keywords:** dental caries, mesenchymal cell, odontoblst, biodentine, regeneration

## Abstract

We developed a novel dentin-pulp-like organoid. It has both stem-cell and odontoblast characteristics using a mesenchymal cell lineage of human dental-pulp stem cells (hDPSCs). The mixture of hDPSCs and Matrigel was transferred into the maintenance medium (MM) and divided into four different groups according to how long they were maintained in the odontogenic differentiation medium (ODM). All organoids were harvested at 21 days and analyzed to find the optimal differentiation condition. To assess the re-fabrication of dentin-pulp-like organoid, after dissociation of the organoids, it was successfully regenerated. Additionally, its biological activity was confirmed by analyzing changes of relevant gene expression and performing a histology analysis after adding Biodentine^®^ into the ODM. The organoid was cultured for 11 days in the ODM (ODM 11) had the most features of both stem cells and differentiated cells (odontoblasts) as confirmed by relevant gene expression and histology analyses. Micro-computed tomography and an electron microscope also showed mineralization and odontoblastic differentiation. Finally, ODM 11 demonstrated a biologically active response to Biodentine^®^ treatment. In conclusion, for the first time, we report the fabrication of a dentin-pulp-like organoid using mesenchymal stem cells. This organoid has potential as a future therapeutic strategy for tooth regeneration.

## 1. Introduction

The dentin and pulp tissue of teeth may be damaged by various causes. Tooth decay is one of the most common causes of dentin and pulp tissue injury throughout the world. Once tooth decay is developed, the teeth become gradually become inflamed. If this inflammation is not controlled, dentin and pulp necrosis will occur eventually, which, in turn, will promote the death of odontoblasts and cause many problems, including malocclusion, masticatory dysfunction, speech disorder, and esthetic problems [1]. Although current treatments with artificial materials for dentin pulp defect and/or enamel defect can restore the esthetic and function of teeth to a certain extent, several complications (such as soft or hard tissue loss, loosening, inflammation/infection with loss of their vitality and sensitivity, chronic pain, and nerve injury following the treatments) remain major limitations for tooth regeneration [2,3,4,5,6,7].

For the last two decades, remarkable breakthroughs in developmental biology and stem-cell biology fields have led to a new level of understanding of how teeth develop and how stem cells can be programmed. Regenerative approaches using biological materials offer a promising strategy to achieve dental structure repair [8]. In tooth or tooth-supporting tissues, various types of stem cells have been identified, including dental-pulp stem cells (DPSCs) [9], periodontal ligament stem cells [10], stem cells from apical papilla [11], and dental follicle cells [12]. Among these, DPSCs of mesenchymal cell lineages that reside in the pulp hold great potential for proliferation. They are known to be differentiated into dentin-forming odontoblasts. They were first isolated and identified from a human impacted third molar in 2000 by Gronthos et al. [13]. Since then, they have been investigated intensely for tooth or dentin regeneration. 

All living beings are configured by their own hierarchical systems, from subcellular level to the whole body. Many research tools have been developed to mimic this organismal hierarchy to address specific questions such as development and pathogenesis. The function of a tissue is determined by both its cellular and its non-cellular compositions. In a cell culture system that resembles in vivo conditions, cells can replicate their behaviors and responses in the body. For this reason, the development of an organoid culture system has become possible.

These days, many studies related to tissue-derived specific adult stem cells in the field of regenerative medicine have been reported. These adult stem cells can self-renew and generate different cell types present in that organ, thereby playing a key role in tissue homeostasis and/or repair. Thus, it is undoubtable that organoids could be cultured from stem cells once sophisticated 3D cell-culture techniques/conditions are employed. Indeed, several types of organoid models have been established, such as stomach [14], pancreas [15], prostate [16,17], liver [18,19], optic cup [20], brain [21], and salivary gland [22].

Most organoids have been targeted to mimic endodermal or ectodermal organs, as described above. On the other hand, studies on organoids for regeneration of dentin or pulp tissue derived from mesoderm layers of the three germ layers are few. Therefore, the objective of this study was to establish a method for fabricating dentin-pulp-like organoids from mesenchymal cells, human dental-pulp stem cells (hDPSCs), and assess their ability to re-organize by in vitro culture. In addition, we evaluated the feasibility of our dentin-pulp-like organoids for drug efficacy testing.

## 2. Materials and Methods

### 2.1. Experimental Design

**Figure 1:**
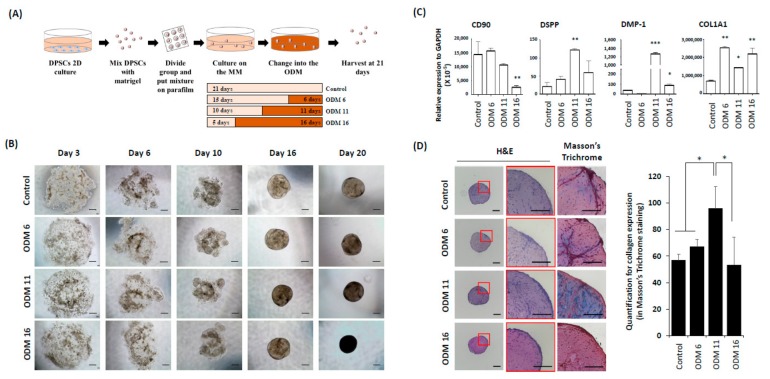
Development of dentin-pulp-like organoid. (**A**) After hDPSCs culture in 2D condition using conventional growth medium, hDPSCs were mixed with Matrigel and adhered onto a parafilm. These mixtures were transferred to organoid MM. To find the optimal culture duration in the ODM, organoids were divided into four different groups according to the exposure duration with the ODM. Finally, these organoids were harvested at 21 days and analyzed. hDPSCs: human dental pulp stem cells, Maintenance medium: MM, ODM: Odontogenic differentiation medium, qRT-PCR: Quantitative real time-polymerase chain reaction, IF: Immunofluorescence analyses, Micro-CT: Micro-computed tomography, EM: Electron microscope. (**B**) On the third day in the maintain media, hDPSCs were dispersed in the Matrigel plug. From the sixth day, hDPSCs started to aggregate gradually. These cells then formed condensed spheroids in all groups from the 16th day. In general, there was no size difference between groups. However, the control and ODM 6 had more lucent spheroids at 20 days than the others. Scale bar = 100 μm, ODM: Odontogenic differentiation medium (**C**) qRT-PCR of each group at 21 days to confirm the undifferentiated cell and odontogenic marker, CD90, DSPP, DMP-,1 and COL1A1. ODM 11 showed increased expression of DSPP and DMP-1. COL1A1 expression was the highest in ODM 6. However, its expression was not significantly higher than that in other OEM groups. All the values are expressed as means ± SDs and normalized to GAPDH expression levels. They were compared with the Kruskall–Wallis test. *, ** and *** indicate *p* < 0.05, *p* < 0.01 and *p* < 0.001, respectively. (**D**) Histological analyses of organoid using H&E and Masson trichrome staining. In ODM 11, a higher cell number was observed in H&E stain, with the strongest collagen staining in Masson’s trichrome staining, although the expression of COL1A1 was not the highest in qRT-PCR. Scale bar = 100 μm. The results of Masson’s trichrome staining were quantified using Image J [23].

Before dentin-pulp-like organoid generation, hDPSCs (Lonza Inc., Basel, Switzerland, PT-5025) were cultured in maintenance medium until passage 5. Next, dentin-pulp-like organoids were fabricated with four different protocols to find the optimal culture duration for the maintenance medium (MM) and odontogenic differentiation medium (ODM). Culture durations for the four groups are shown in Figure 1A. All organoids were observed under a light microscope and harvested at 21 days. Their characteristics were analyzed by a quantitative real-time polymerase chain reaction (qRT-PCR), immunofluorescence (IF), and histology. After optimization of culture and differentiation, micro-compute tomography (micro-CT) and electron microscopy (EM) were performed for structural analyses. Next, organoids were dissociated and reorganized into dentin-pulp-like organoids to evaluate their forming structure ability. We also assessed their biologic activities after adding Biodentine^®^ (FDA-approved commercially available stimulator of odontoblastic differentiation) during organoid culture and differentiation.

### 2.2. Culture of Dentin-Pulp-Like Organoids

First, hDPSCs were purchased from Lonza and cultured in the MM which consisted of a minimum of essential medium α, nucleosides (Gibco, Grand Island, NY, USA), 10% fetal bovine serum (FBS, Gibco), and 1% penicillin-streptomycin (PS, Welgene, Daegu, Korea). When cell confluency was about 70%–80%, cells were detached using trypsin ethylenediaminetetraacetic acid (trypsin EDTA, Gibco). The 5 × 10^4^ cells/10 μL of medium was then mixed with Matrigel (BD Biosciences, San Jose, CA, USA) at a ratio of 1:1, plated onto the parafilm, and incubated with 5% CO_2_ at 37 °C for 30 min for polymerization of matrices. Constructs were cultured in the MM and ODM which consisted of Dulbecco’s Modified Eagle’s Medium 1x (DMEM, Welgene), 10% FBS (Gibco), 1% PS (Welgene), 50 μM L-ascorbic acid (Sigma, St. Louis, MO, USA), 10 mM β-glycerophospate (Sigma), and 100 nM dexamethasone (Peprotech, Rocky Hill, NJ, USA).

### 2.3. Total RNA Extraction and qRT-PCR

Total RNA was isolated from cells as described previously [24]. Briefly, RNA was extracted using a MagListo™ 5M Cell Total RNA Extraction Kit (Bioneer, Daejeon, Korea, K-3611). Then cDNA was synthesized using the extracted total RNA and a PrimeScript™ RT Master Mix (Perfect Real Time, TAKARA, RR036A). Quantitative RT-PCR was performed with a Thermal Cycler Dice^®^ Real Time System III (Takara) using SYBR^®^ Premix Ex Taq™ II (TaKaRa, Shiga, Japan, RR820A). The sequences of the PCR primer are listed in Table 1. The experiments were carried out in triplicate.

### 2.4. Histology and Immunofluorescence

Organoids of each groups were washed with Dulbecco’s phosphate buffered saline (D-PBS), fixed with 4% paraformaldehyde (Biosolution), and embedded in paraffin. Sample slices of 4–6 μm in thickness were deparaffinized in xylene and dehydrated in a graded series of ethanol. These samples were then treated with hematoxylin (Sigma, St. Louis, MO, USA, H3136), eosin Y (Sigma, St. Louis, MO, USA, HT110180), and Masson’s trichrome staining kit (DAKO, Carpinteria, CA, USA, QAR17392-2) according to their respective manufacturers’ protocols [25]. For the immunofluorescence analysis, samples were treated by 5% normal goat serum (Vector) in Tris-buffered saline (Welgene) at room temperature for 2 h, incubated with primary antibodies at 4 °C overnight, and then reacted with secondary antibodies at room temperature for 2 h. Antibodies used for immunostaining were CD90 (Abcam, Cambridge, UK, ab181469), DMP-1 (Santa Cruz, Dallas, TX, USA, sc-73633), NGFR (Santa Cruz, Dallas, TX, USA, sc-13577) and PHEX (Lsbio, Seattle, WA, USA, c681043-100).

### 2.5. Analysis Using Micro-CT

After 21 days of 3D organoid culture using MM and/or ODM, mineralization of the organoid was evaluated by micro-CT. Briefly, fabricated organoids were fixed with 4% paraformaldehyde and scanned using a high-resolution micro-CT (SkyScan 1176, Bruker MicroCT N.V., Kontich) at an image resolution of 5 µm with the following settings: 0.5 mm of aluminum filter, X-ray voltage of 50 kVp, anode current of 500 mA, exposure time of 250 ms, frame averaging of two, and rotation step of 0.4 degrees. 3D images from 2D X-ray projections were then reconstructed by implementing the Feldkamp algorithm. NRecon reconstruction software and CT-Analyzer software (SkyScan 1172) were used for appropriate image corrections and 3D morphometric analyses, respectively. The volume of interest was defined as 1.5 mm in diameter and height to include the whole organoid of each group. The volume and percent of mineralization were analyzed using CT-Analyzer software as described previously [26,27].

### 2.6. Electron Microscopic Analysis

To detect characteristics of differentiated odontoblast-like cells on organoids, a scanning electron microscope (SEM) and a transmission electron microscope (TEM) were used. Briefly, cultured organoids were washed with D-PBS and fixed with 2% glutaraldehyde-paraformaldehyde in 0.1 M phosphate buffer (PB, pH 7.4) for 12 h. After washing with 0.1 M PB, samples were post-fixed with 1% OsO4 dissolved in 0.1 M PB for 2 h, dehydrated in an ascending gradual series (50%–100%) of ethanol, infiltrated with propylene oxide, and embedded by Poly/Bed 812 kit (Polysciences, Washington, PA, USA). After pure fresh resin embedding and polymerization at 65 °C in an electron microscope oven (DOSAKA) for 24 h, they were initially cut into about 200–50 nm-thick sections and stained with toluidine blue (Sigma) for examination under a light microscope. Some 70-nm thin sections were double-stained with 6% uranyl acetate and lead citrate (Thermo Fisher Scientific, Waltham, MA, USA) for contrast staining. These sections were cut with a Leica EM UC-7 (Leica Microsystems, Tokyo, Japan) equipped with a diamond knife (Diatome, Hatfield, PA, USA) and transferred onto copper and nickel grids. All the thin sections were observed with a TEM (JEOL) at an acceleration voltage of 80 kV.

### 2.7. Statistical Analyses

Statistically significant differences were analyzed using SPSS for Windows Version 18.0 (SPSS, Chicago, IL, USA). Significance was considered at *p* < 0.05. All the experiments were conducted at least three times. Means and standard deviations were calculated from numerical data and presented in the text, figures, and figure legends. In the figures, bar graphs and error bars represent means and one standard deviation in each. Means of more than two groups were compared by the Kruskal–Wallis test with post hoc tests of Bonferoni. The Mann–Whitney U test was also used to compare differences between two data sets.

## 3. Results

### 3.1. Progression of Dentin-Pulp-Like Organoids from Human Dental-Pulp Stem Cells (hDPSCs)

The development of organoids was observed under a light microscope. After 3 days of culturing in the maintenance medium (MM), hDPSCs were dispersed in the Matrigel plug (Figure 1B). hDPSCs of all groups started to aggregate gradually at Day 6 and formed condensed spheroids Day 16. All organoids had sizes from 150 µm to 250 μm. In particular, the lucent area inside the organoid was observed in control and ODM 6 groups.

### 3.2. Organoids of ODM 11 Have the Highest Differentiation Potential While Preserving Stem-Cell Characteristics

**Figure 2:**
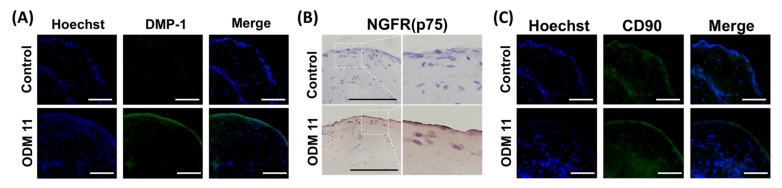
Expression of odontogenic and stem-cell markers in ODM 11 group. Expression of DMP-1 (**A**), NGFR (**B**) and CD90 (**C**) in organoids of control and ODM 11 groups. Blue represents the staining of cell DNA with Hoechst. As expected, the ODM 11 group showed positive staining for DMP-1, NGFR and CD90, but the intensity of CD90 staining was lower in ODM 11 than in the control. The panels show merged confocal images of Hoechst and secondary. Scale bar = 100 μm.

In organoids of the ODM 11 group, mRNA expression levels of *DSPP* and *DMP-1*, two specific markers of odontogenic differentiation, were significantly induced (*p* < 0.01 and *p* < 0.001, respectively). In addition, in the ODM6 and ODM 11 groups, the expression of CD90 that indicated the preservation of undifferentiated cell properties was not significantly different compared to that of control group (*p* > 0.05). *COL1A1* expression was the highest in the ODM 6 group. However, it was not significantly different from that in the ODM 11 group (Figure 1C). Histologically, after H & E staining, the higher cell number was observed in the ODM 11 group. This group also had the strongest expression of collagen, a major protein component of dentin to indicate the mineralization in Masson trichrome staining, although the mRNA expression level of *COL1A1* was not the highest in qRT-PCR. In contrast, there was no staining for collagen in the control (Figure 1D). In the result of immunostaining for relevant protein expression, the organoid in the ODM 11 group showed positive staining for DMP-1, NGFR and CD90 proteins, although the intensity of CD90 was lower than that in the control group (Figure 2).

### 3.3. Imaging Evaluation Using Micro-CT and EM Demonstrates Mineralization and Odontoblastic Differentiation in ODM 11

**Figure 3:**
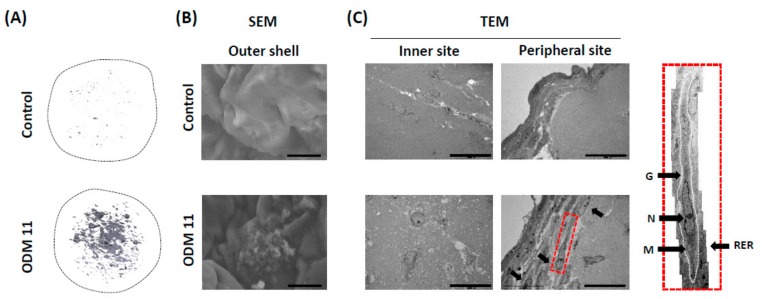
Imaging evaluation using micro-CT and EM demonstrates mineralization and odontoblastic differentiation in ODM 11. (**A**) Representative images of Micro-CT revealing marked mineralization in ODM 11. However, only a scanty amount of mineralization was shown in the control. (**B**) SEM images revealed the calcified matrix on the surface of organoids with tubule. (**C**) TEM image showed odontoblast-like cells with an elongated shape. These cells were located mostly in the peripheral area. In the peripheral area, odontoblast-like cells had proper cellular structures. Scale bar = 10 μm for SEM and 20 μm for TEM. Micro-CT: Micro-compute tomography; EM: Electron microscope; SEM: Scanning electron microscope; TEM: Transmission electron microscope; G: Golgi apparatus; N: Nucleolus; M: Mitochondria.

To evaluate the typical characteristics of stem cells and odontoblastic differentiated cells in the organoid of the ODM 11 group, structural changes of each organoid were analyzed using a micro-CT and EM scanning for selected samples. Micro-CT observations correlated well with histologic analyses. Robust mineralization was founded in ODM 11. However, only a scanty amount of mineralization was observed in the control (Figure 3A). Next, scanning electron microscope images revealed calcified matrix on the surface of organoid with tubule (Figure 3B). More importantly, the transmission electron microscope images show odontoblast-like cells with an elongated shape and proper cell structures, including nucleolus (N), golgi apparatus (G), rough endoplasmic reticulum (RER), and mitochondria (M) as previously reported [28]. They were located mainly in the peripheral area, not in the central zone (Figure 3C).

### 3.4. Dissociated and Refabricated Organoids of ODM 11 Have Viable Cells

**Figure 4:**
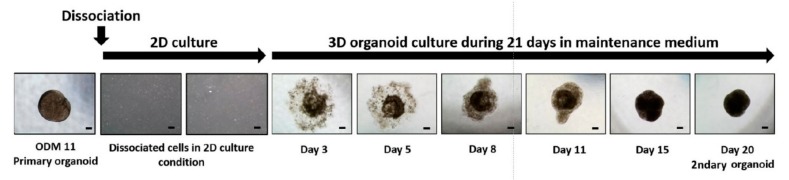
Dissociation and refabrication of organoid confirmed that ODM 11 consisted of viable cells. Primary dentin-pulp-like organoid from ODM 11 was dissociated using trypsin EDTA. After 12 days of culture, cells were cultured again using organoid MM. During primary dentin-pulp-like organoid fabrication, dissociated cells started to aggregate and formed the organoid at 21 days again. Representative images were taken under a light microscope. Scale bar = 100 μm, MM: maintenance medium.

ODM 11 organoids were dissociated using trypsin EDTA and reassembled in MM condition because the fabricated organoid should have viable cells to make it right. Briefly, dissociated cells were cultured with MM for 12 days. Cells were refabricated into organoids using MM only. As shown in the primary organoid previously, dissociated cells started to aggregate and formed the organoid at 21 days, indicating that cells from the ODM 11 organoid were viable (Figure 4). To confirm differences in odontogenic differentiation between ODM 11 primary organoid and ODM 11 secondary organoid, the organoids of two group were additionally analysis by immunofluorescent for DMP-1. And, we observed similarity to expression of DMP-1, between primary ODM11 and secondary ODM11 (data not shown). Undifferentiated characters of dissociated cells were confirmed by FACS analysis for stem cell surface marker (Figure S1).

### 3.5. Biodentine^®^ Supplements Further Enhance Stimulation of ODM 11 Differentiation

**Figure 5:**
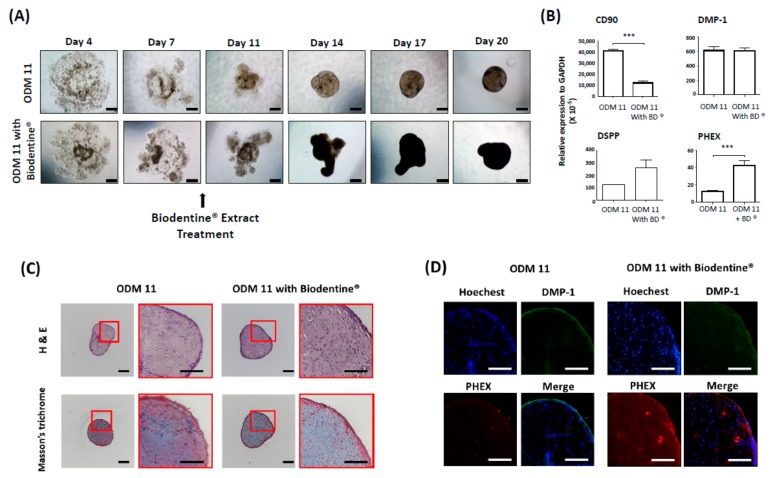
ODM 11 demonstrated biologically active response to Biodentine^®^ extract treatment. (**A**) During the fabrication of ODM 11, 40 mg/mL of Biodentine^®^, which was previously reported to enhance odontogenic differentiation and approved by the US FDA, was additionally added into the ODM. Under a light microscope examination, faster cell aggregation and more condensed organoid formation were detected compared to ODM 11. Scale bar = 100 μm. (**B**) In the Biodentine^®^ exposure group, CD90 expression level was significantly decreased, while the expression level of PHEX, a terminal differentiation marker of odontoblast, expression, was significantly increased. *** indicate *p* < 0.05 and *p* < 0.001, respectively. (**C**) Biodentine^®^ increased collagen formation, which was assessed by Masson’s trichrome staining. Scale bar = 100 μm. (**D**) Positive immunofluorescence staining for DMP-1 and PHEX. Magnification: 40×.

Because the organoid should recapitulate the organ and need to be biologically active as much as possible, we evaluated responses of the ODM 11 after Biodentine^®^ application. The results demonstrate that the organoid with Biodentine^®^ had faster cell aggregation. Irregularly shaped organoid had a deeper dense color than ODM 11 without Biodentine^®^. The mRNA expression level of *PHEX*, a late differentiation maker of odontoblasts, was significantly greater in ODM 11 with Biodentine^®^. Furthermore, Biodentine^®^ increased collagen formation, which was assessed by Masson’s trichrome staining. There was a positive IF staining for DMP-1 and PHEX, although there was no statistically significant difference in *DMP-1* gene expression between ODM 11 without Biodentine^®^ and ODM 11 with Biodentine^®^ (Figure 5).

## 4. Discussion

Stemming from in vitro and in vivo pre-clinical and human models, tissue-engineering-based strategies continue to demonstrate great potential for the regeneration of dentin-pulp tissue, particularly for necrotic, immature, and permanent teeth. However, pure stem cells are known to only have limited efficacy. In particular, hard dental tissue structure such as dentin and tooth is fairly challenging to regenerate since it relies on the presence of odontoblasts [29]. By manipulating culture conditions or methods in which stem cells differentiate, it is possible to control and boost differentiation pathways and generate cultures enriched in odontoblast-like precursors in vitro. Several reports have shown that isolated pulp cells can be induced to differentiate into odontoblast-like cells and generate a dentin-like mineral structure [29]. However, most of previous analyses were based on cells of 2D cultures.

Previously, several different protocols to generate organoids have been reported [30,31,32,33]. However, currently, most organoids are made for recapitulating organs originating from the ectoderm or the endoderm using embryonic stem cells or induced pluripotent stem cells [34].

Regarding mesenchymal lineage cells, Thomas et al. have reported that the spheroid or 3D cell aggregate culture has a much better anti-inflammatory effect than did human mesenchymal stem cells by the adherent culture technique [32,35]. However, the spheroid only has stem cells. It does not have relevant cells present in vivo such as odontoblasts in dentin-pulp tissue. Although tissue explants or slices might be alternatives for organoids, they could only transiently capture physiologically relevant cell organization and interactions. In addition, they tend to quickly lose their phenotype. Moreover, it is difficult to maintain them for extended periods of time [36]. Other 3D culture systems have also been proposed. However, they often lack the presence of the relevant stem or progenitor cell populations required to sustain the 3D culture. Thus, they lack the ability to maintain cells with the capacity for self-renewal and differentiation [37].

In this study, we tried to generate dentin-pulp-like organoid using a floating culture method for mesenchymal lineage cells, hDPSCs, under different culture conditions. Our dentin-pulp-like organoid demonstrated characteristic markers of both stem cells and differentiated odontoblast-like cells. Imaging analyses using micro-CT showed mineralization in the ODM 11 group. EM images also demonstrated the typical shape of stem cells and odontoblasts with spatial arrangement. Odontoblasts were mainly found in the outer portion of the organoid with stem cells in the inner side. The ability of reorganization after dissociation and proper biological responses of organoids were demonstrated.

Before ODM was tried in this study, we were concerned about an ideal culture medium for odontogenic differentiation because there was no established protocol for odontogenic organoid culture, especially for dentin or pulp tissue. We tried to find the optimal duration of differentiation and found that ten days in the MM were long enough to stabilize and prepare for differentiation. The organoid from the ODM 11 group had the highest expression of both stem-cell and odontoblast markers. They also had a better spatial arrangement than other groups. We speculate that the number of cells and the ratio between differentiated cells and progenitor cells might have affect cell–cell interactions that are crucial for dentin-pulp-like organoid development.

We applied the Biodentine^®^ extract on the ODM and analyzed responses of the dentin-pulp-like organoid. The Biodentine^®^ (Septodont, St.Maur-des-Fossés, France) is a new bioactive cement based on calcium silicate that has been recently launched in the dental market as a ‘dentin substitute’ [38]. Luo Z et al. have studied the effect of Biodentine^®^ on hDPSCs and found that it can significantly increase the proliferation, migration, and adhesion of stem cells when it is placed directly in contact with the pulp [39]. This further reflects the bioactivity and biocompatibility of the material. It can also promote mineralization, generating a reactionary dentin or a dense dentin bridge when it is placed in contact with pulp. As expected, we found that CD90 expression was significantly decreased in the Biodentine^®^ exposure group. However, terminal differentiation markers of odontoblast PHEX expression were significantly increased. Biodentine^®^ also increased collagen formation and staining of DMP-1 and PHEX protein in IF. Collectively, these results suggest that dentin-pulp-like organoids not only provide potential advantages of adopting a biological tissue-engineering strategy for dentin-pulp tissue regeneration, but also might be applicable for drug screening of tooth regeneration like other types of organoid. They can also be used for research on tooth development.

We acknowledge that our experiment has several limitations. Although we generated viable organoids that preserved dentin-pulp characteristics using human stem cells, there was still a profound obstacle in their clinical application. First, we could not prove an in vivo dentin-pulp tissue regeneration effect using dentin-pulp-like organoids. Second, during the fabrication of the organoid in this study, we mixed hDPSCs with Matrigel which was derived from Engelbreth-Holm-Swarm mouse sarcoma cells, thus hindering a clinical trial [28,40]. In vivo efficacy tests are warranted in the future using alternative materials instead of Matrigel to generate dentin-pulp-like organoids. However, such organoids should be useful for screening candidate drug materials efficiently.

In conclusion, using hDPSCs, we generated dentin-pulp-like organoids containing relevant cells and proteins. Such organoids that could support viable cells were successfully reconstructed after dissociation. They showed proper responses to a biological stimulator. Such dentin-pulp-like organoids have potential as a novel research tool with possible applications in regenerative medicine in the future.

## Figures and Tables

**Table 1:** The primers used for quantitative RT-PCR.

Gene	Strand	Primer Sequences	Product Size
DMP-1	Forward	TTGACAATGAGGACCGGGTG	171 bp
	Reverse	TCCTGATGCTCTCTGGGTCA	
DSPP	Forward	TGAGGATGTCGCTGTTGTCC	188 bp
	Reverse	CTTCTCCAGTGCCTGGTGTT	
COL1A1	Forward	AGTGGTTTGGATGGTGCCAA	170 bp
	Reverse	GCACCATCATTTCCACGAGC	
CD90	Forward	CAGCATCGCTCTCCTGCTAA	134 bp
	Reverse	ACTGGATGGGTGAACTGCTG	
PHEX	Forward	GTTCTGGGCACGATCCTCTT	164 bp
	Reverse	TCACAAGCGAACCGGAAGAA	

## Supplementary Materials

The following are available online at https://www.mdpi.com/2073-4409/9/3/642/s1, Figure S1: FACS analysis of stem cells marker in the cells from dissociated organoids.
