# Supplementary material for: Fabrication of Dentin-Pulp-Like Organoids Using Dental-Pulp Stem Cells
Source: Cells. 2020 Mar 6;9(3):642. doi: 10.3390/cells9030642 (PMC7140482; doi:10.3390/cells9030642)
Supplement: Supplementary file 1 [file cells-09-00642-s001.pdf]

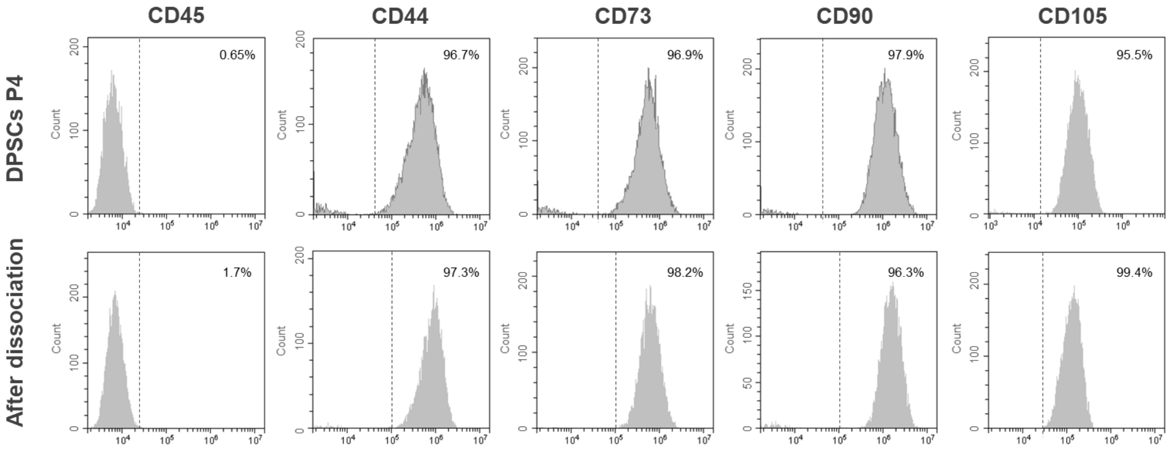

Figure S1. FACS analysis of stem cells marker in the cells from dissociated spheroids. Primary dentin-pulp-like spheroids from ODM 11 were dissociated using trypsin EDTA. To confirm the undifferentiated cell population in the spheroids, cells were analyzed by FACS and compared with P4 DPSCs. Briefly, cells were incubated in the blocking solution (1 % BSA in PBS) for 30 min after PBS washing. And then, cells were incubated with antibodies (CD45, CD44, CD73, CD90-FITC, and CD105-PE) for 1 hour in the RT, and analyzed by FACS. The cell surface markers were purchased from BD Bioscience.
